# Diosgenone Synthesis, Anti-Malarial Activity and QSAR of Analogues of This Natural Product

**DOI:** 10.3390/molecules18033356

**Published:** 2013-03-14

**Authors:** Adriana Pabón, Gustavo Escobar, Esteban Vargas, Víctor Cruz, Rafael Notario, Silvia Blair, Fernando Echeverri

**Affiliations:** 1 Grupo Malaria, Facultad de Medicina, Universidad de Antioquia, 050010 Medellín, Colombia; E-Mails: apabon72@gmail.com (A.P.); silviablair@gmail.com (S.B.); 2 Programa de Biología, Facultad de Ciencias Básicas, Universidad del Atlántico, 080001 Barranquilla, Colombia; 3 Grupo de Química Orgánica de Productos Naturales, Instituto de Química, Universidad de Antioquia, 050010 Medellín, Colombia; E-Mails: gescobar104@yahoo.es (G.E.); estebanvargas7@gmail.com (E.V.); 4 Instituto de Estructura de la Materia, CSIC, 28006 Madrid, Spain; E-Mail: victor.cruz@iem.cfmac.csic.es; 5 Instituto de Química-Física Rocasolano, CSIC, 28006 Madrid, Spain; E-Mail: rnotario@iqfr.csic.es

**Keywords:** malaria, natural product, steroid, diosgenone, structural analogue, animal model

## Abstract

*Solanum nudum* Dunal steroids have been reported as being antimalarial compounds; however, their concentration in plants is low, meaning that the species could be threatened by over-harvesting for this purpose. Swern oxidation was used for hemisynthesis of diosgenone (one of the most active steroidal sapogenin diosgenin compounds). Eighteen structural analogues were prepared; three of them were found to be more active than diosgenone (IC_50_ 27.9 μM *vs.* 10.1 μM, 2.9 μM and 11.3 μM). The presence of a 4-en-3-one grouping in the A-ring of the compounds seems to be indispensable for antiplasmodial activity; progesterone (having the same functional group in the steroid A-ring) has also displayed antiplasmodial activity. Quantitative correlations between molecular structure and bioactivity were thus explored in diosgenone and several derivatives using well-established 3D-QSAR techniques. The models showed that combining electrostatic (70%) and steric (30%) fields can explain most variance regarding compound activity. Malarial parasitemia in mice became reduced by oral administration of two diosgenone derivatives.

## 1. Introduction

Malaria is a parasitic disease which is responsible for about one million deaths annually [1]; its high morbidity and mortality affects more than 106 countries, in which there were nearly 216 million episodes of malaria in 2010 alone. There is a compelling need to look for new strategies for treating malaria [2] considering the above and first-line drugs’ high failure rates (*i.e.*, chloroquine, amodiaquine, sulfadoxine/pyrimethamine) [3]; therefore compounds having new mechanisms of action and less (or no) adverse effects are urgently needed. It is well-recognized that natural products represent a source of new drugs and several properties, including flavonoids, alkaloids (*i.e.*, quinine), coumarins, quinines, terpenes and lactones (*i.e.*, artemisinins) [4].

**Figure 1:**
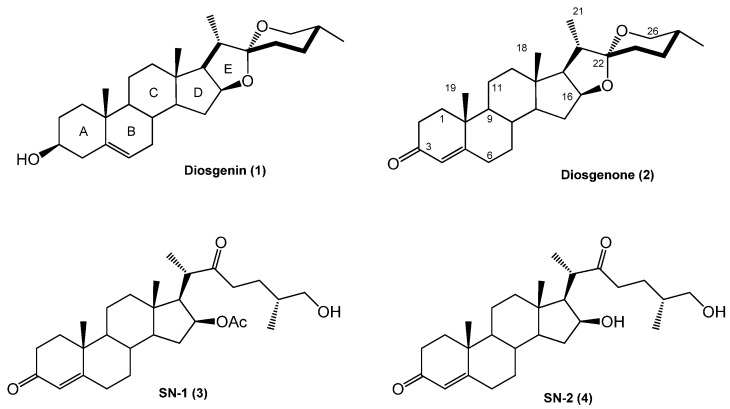
Structure of diosgenin and diosgenone, SN-1 and SN-2 *S. nudum* sapogenin.

It has been reported that diosgenone (**2**) and several steroid derivatives isolated from *Solanum nudum* Dunal having a 4-en-3-one system, such as SN-1 (**3**) and SN-2 (**4**) (Figure 1), have antiplasmodial activity against FcB-2 chloroquine-resistant strains [5,6,7]. Only a few reports concerning antiplasmodial activity of steroids could be found in the literature, in spite of the fact that some steroidal alkaloids are active against malaria [8].

*S. nudum* steroidal compounds’ antiplasmodial activity and their lack of mutagenicity, clastogenicity [9,10] and cytotoxicity [11] have substantiated this plant’s potential use in anti-malarial medication and encouraged attempts to search for new pharmacophores and more active compounds than diosgenone. The presence of an enone structure in the A-ring and a spirostane in the E-ring were thus select to generate a series of derivatives as potential antimalarial products. However, diosgenone is only present in the plant in terms of a few milligrams per kilogram of plant material; obtaining it by hemisynthesis from commercial diosgenin was thus deemed reasonably logical and would also lead to obtaining a further series of derivatives.

Natural compounds’ synthesis is complex due to the presence of several functional groups in the side chain and D-ring; diosgenone hemisynthesis from diosgenin was thus a sound alternative for determining its structure-activity relationship and carrying out studies of its *in vivo* activity, absorption and toxicity. Since the synthesis of other compounds involving only diosgenone A- and B-rings is expected to yield new types of antimalarial substances, diosgenone and some diosgenin steroid derivatives were obtained and their antiplasmodial potential determined.

A preliminary structure-activity relationship was established and quantitative structure-activity relationship (QSAR) studies determined correlations between molecular structure and biological activity. This involved using 3D quantitative structure activity relationship (3D-QSAR) [12], a well-established drug design methodology providing information regarding statistical correlations between molecular structure descriptors and experimentally-measured bioactivity [13]. The partial linear squares (PLS) [14] algorithm provided a solid mathematical background for 3D-QSAR for statistically-significant models having acceptable predictive capability, ensured by very conservative cross-validation testing. This chemometric tool has been used for studying the leishmanicidal activity of some acnistins and withajardins [15]. 3D-QSAR models have provided detailed structural information about the factors best explaining the variance observed in experimental bioactivity data.

## 2. Results and Discussion

### 2.1. Chemical Transformation

Diosgenone (**2**) was obtained from diosgenin (**1**) through a Swern oxidation [16]; the double bond was then isomerized with oxalic acid in a two-step procedure in a one-pot reaction (84%). This type of reaction has been reported for other compounds and resembles the primary transformation of steroidal sapogenins into progesterone [17].

**Scheme 1:**
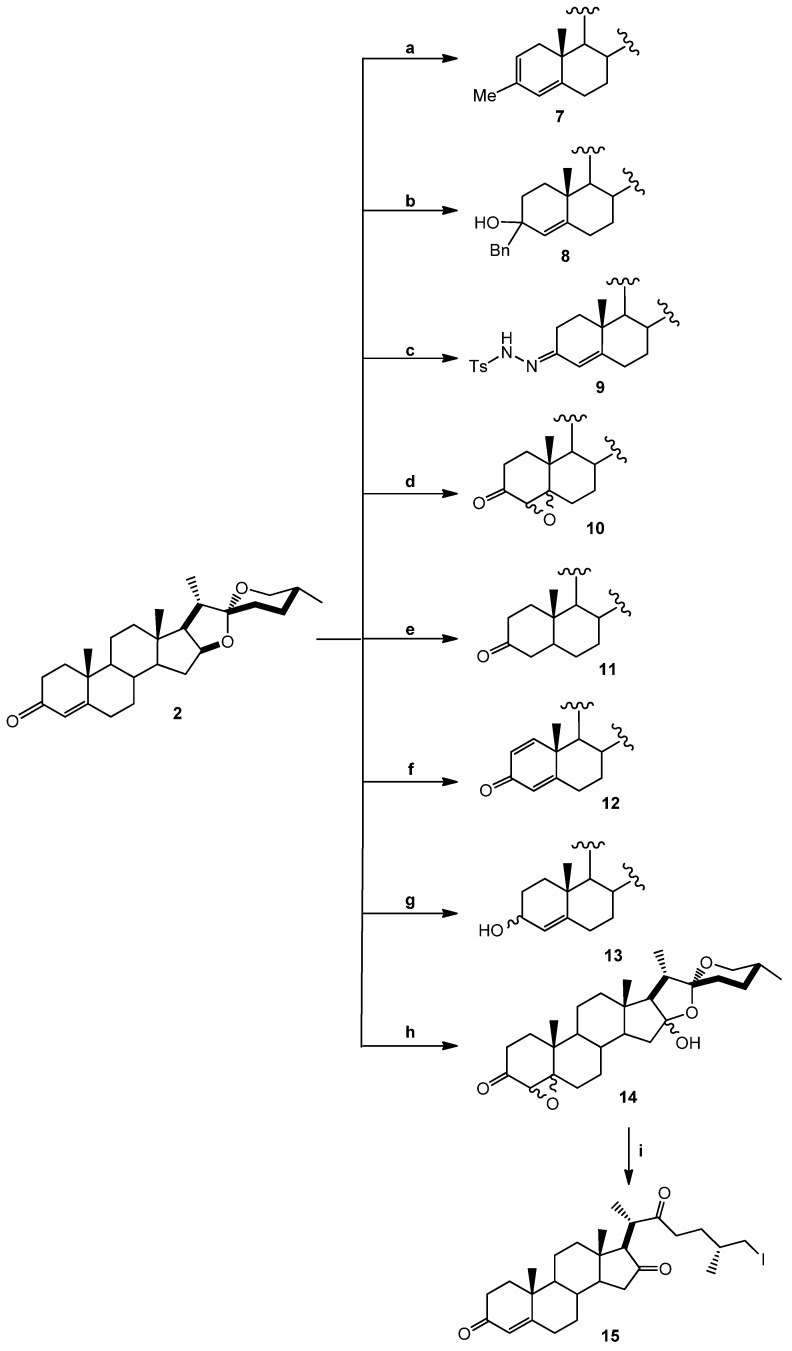
Diosgenone (**2**) and derivatives **7**–**15** prepared by modifications made in the A- and B-rings and the spiroketal system.

Nine diosgenone analogues (compounds **7**–**15**) and five analogues of diosgenin (compounds **16**–**20**) were synthesized (Scheme 1). These fourteen derivatives were synthesized through several structural modifications to the A- and B-rings and in the diosgenin (**1**) and diosgenone (**2**) spiroketal system.

**Scheme 2:**
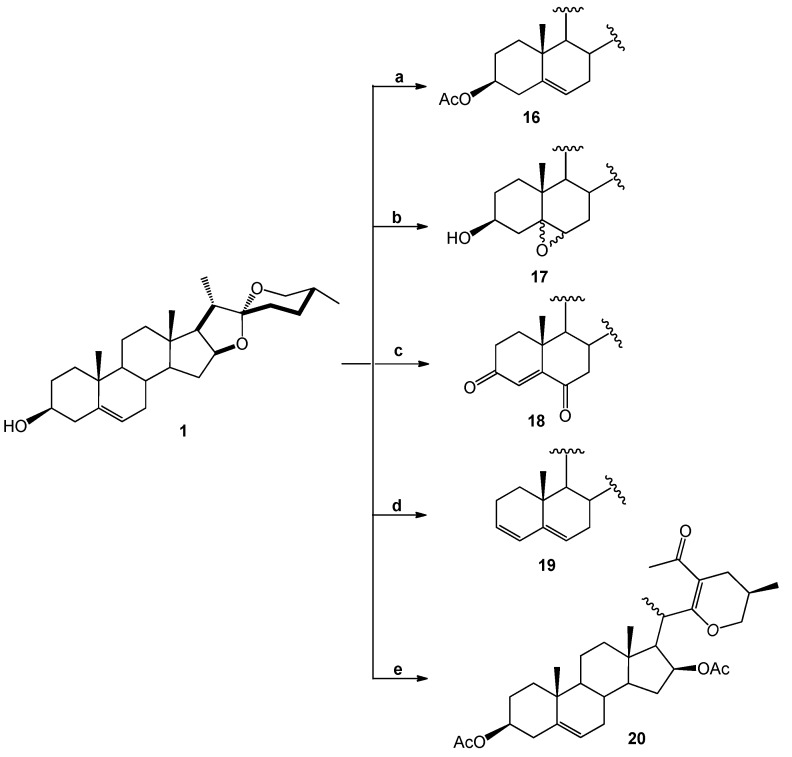
Diosgenin derivatives **16**–**20** obtained by modifications made to the A- and B-rings. Diosgenin (**1**).

Derivatives **7** and **8** were prepared by adding Grignard reagents (Me, Bn, Scheme 2) to compound **2**; compound **9** was produced by adding *p*-toluenesulfonic hydrazine. Treating the double bond with H_2_O_2_ produced compound **10** as a non-isolable 2:1 mixture (according to NMR spectra) of α and β-epoxides. Hydrogenation in Pd/C and reduction with NaBH_4_ yielded compounds **11** and **13**. Compound **12** was obtained from **2** with an excess of 2,3-dichloro-5,6-dicyanobenzoquinone (DDQ) in refluxing toluene, while Oxone^®^ (potassium peroxymonosulfate) treatment of **2** in basic media produced **14**, in turn transformed into **15** with TMSI.

Five derivatives were also prepared from diosgenin **1**; Compound **16** was obtained with acetic anhydride and epoxidation with H_2_O_2_ produced compound **17**, while treatment with excess PCC produced **18** and a Clemmensen reduction yielded **19**. Derivative **20** was produced after treating **1** with acetic anhydride and zinc chloride [18] (Scheme 2).

**Scheme 3:**
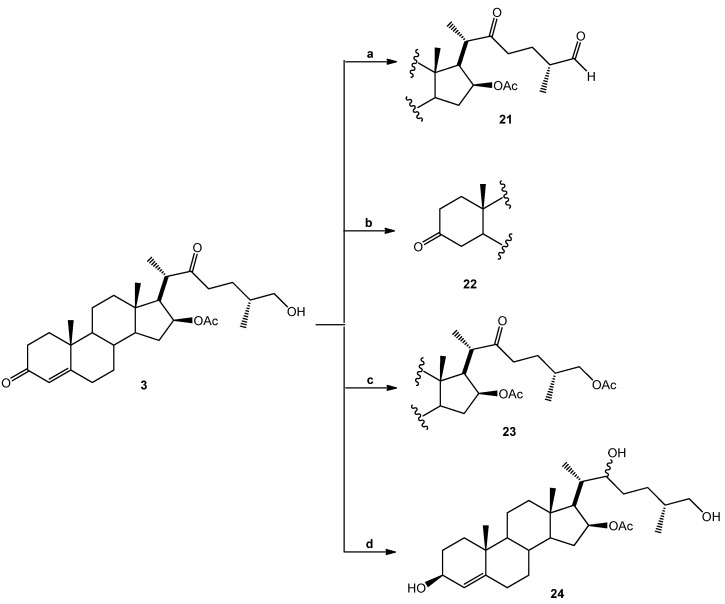
SN-1 derivatives **21**–**24** produced by modifications made to the side chain and A-ring. SN-1 (**3**).

Four other derivatives were prepared from compound SN-1 (**3**). Compound **21** was obtained by Swern oxidation, **22** through a hydrogenolysis reaction with Pd/C as catalyst, while **23** was the product of acetylation with excess acetic anhydride and **24** was obtained after reduction with NaBH_4_ in methanol at room temperature, unfortunately it was not possible to assign the diastereoisomeric ratio (Scheme 3). Derivative **25** was produced from SN-2 (**4**) with another Swern oxidation.

### 2.2. Antiplasmodial Activity

The anti-plasmodium activity of 25 derivatives, including progesterone (**5**) and 17-OH progesterone (**6**), was assayed in two *P. falciparum* strains (Table 1); this analysis was based on the dose inhibiting 50% of parasite growth (IC_50_) expressed in the FcB2 (chloroquine-resistant) and NF-54 *P. falciparum* strains (sensitive to chloroquine, amodiaquine, quinine and artesunate but mefloquine-resistant).

**Table 1:** *In vitro* biological activity of derivatives regarding *Plasmodium falciparum* FCB-2 and NF-54 strains and on HepG2A (human hepatoma cell line).

Compound		IC_50_ μM ± SD		RI *	CC_50_ μM	SI **
		FCB-2	NF-54			
1	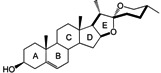	>482.4	>482.4	1.0	1111.4	2.3
2	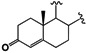	27.9 ± 5.5	35.4 ± 2.2	0.8	265.1	9.5
3	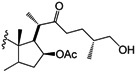	21.35 ± 0.2	32.96 ± 0.8	0.6	60.00	2.8
4	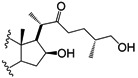	222.8 ± 12.3	248.0 ± 5.3	0.9	2145.7	9.6
5	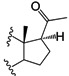	38.2 ± 1.2	25.4 ± 1.6	1.5	193.0	5.0
6	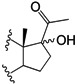	>302.6	>302.6	1.0	>1145	3.8
7	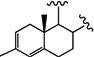	70.4 ± 5.5	34.6 ± 3.3	2.0	216.7	3.1
8	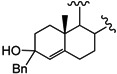	>396	>396	1.0	>1981	7.8
9	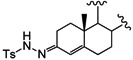	56.1 ± 0.3	52.5 ± 7.7	1.1	>1166.6	30.7
10	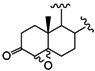	56.7 ± 6.3	105.7 ± 7.7	0.5	100.6	1.8
11	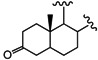	>482.4	>482.4	1.0	>916.3	1.9
12	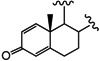	36.0 ± 2.3	29.2 ± 1.1	1.2	>487	6.6
13	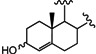	63.2 ± 0.3	71.9 ± 0.3	0.9	>2411	38.2
14	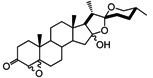	10.1 ± 1.3	8.5 ± 2.3	1.2	153.4	15.2
15	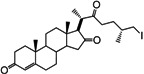	2.9 ± 0.3	2.2 ± 0.4	1.3	90.2	31.1
16	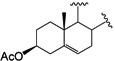	>438.0	>438.0	1.0	>2,189.9	5.0
17	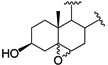	>409.3	183.8	2.2	>2,046.5	5.0
18	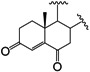	11.3 ± 1.6	9.8 ± 1.0	1.2	191.5	16.9
19	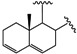	438.2 ± 12.0	369.6 ± 8.3	1.2	>2,521.4	6.5
20	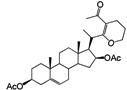	>379.7	>379.7	1.0	>1,898.6	5.0
21	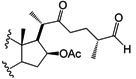	92.2 ± 12.3	70.8 ± 0.8	1.3	424.3	4.6
22	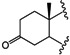	94.2 ± 5.3	62.1 ± 8.9	1.5	82.4	0.9
23	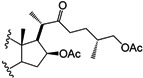	17.3 ± 1.4	0.6 ± 0.9	28.8	280.8	16.2
24	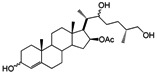	95.0 ± 7.3	100.9 ± 9.9	0.9	306.5	3.2
25	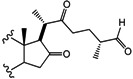	122.6 ± 12.8	95.9± 8.3	1.3	45.2	0.4
CQ	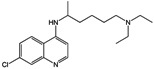	0.157 ± 0.01	0.02 ± 0.005	7.9	97.2	486.0
AF-B	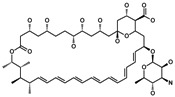				39.6	

* RI (resistance index) = IC_50_ FCB-2 (resistant strain)/IC_50_ NF-54 (sensitive strain); ** SI (selectivity index) = CC_50_ HepG2/IC_50_ parasite FCB-2; CQ: chloroquine (control of antiplasmodial assays); AF-B: amphotericin B (positive control of cytotoxicity assays).

Table 1 shows IC_50_, CC_50_ (50% cytotoxic dose in HepG2-A16 cells), selectivity index (SI = IC_50_/CC_50_) and resistance index values (RI = IC_50_ FCB-2/IC_50_ NF-54) for all compounds. Chloroquine had IC_50_ 157.9 nM in the FCB-2 strain and IC_50_ 20 nM in the NF-54 strain. Diosgenin (**1**) lacked antiplasmodial activity since its IC_50_ was >482.4 µM against *P. falciparum*; however, diosgenone (**2**) was active, having IC_50_ 27.9 µM in the FcB-2 strain and 35.4 µM IC_50_ in the NF-54 strain.

Compounds **7**, **9**, **10** and **13** had marginal activity compared to diosgenone and **12** had similar activity to diosgenone, though derivatives **8** and **11** were inactive against both *P. falciparum* strains. Compounds **14** and **15** had high antiplasmodial activity (IC_50_ 10.1 µM and 2.9 µM, respectively), and high SI (15.2 and 31.1, respectively). Compounds **16**, **17**, **19** and **20** were neither active nor toxic. However, more active compound **15** was very unstable since solution color in NMR tube changed from clear to deep brown, so its effect on *P. berghei* was not evaluated (iodine being generated during decomposition may have been responsible for such activity). Regarding RI, it was found that derivatives **2**, **3**, **4**, and **10** had values below zero, indicating better antiplasmodial activity in chloroquine-resistant parasites.

The importance of an α,β−unsaturated system in the molecules’ A-rings (exhibiting antiplasmodial activity) was demonstrated when the 3-en-1-one system was modified in diosgenone by hydrogenation or reduction (derivatives **7**–**13**, but not **12**). Such a system is present in progesterone **5**, a compound exhibiting antiplasmodial action (IC_50_ 38.2 µM), but lacking a spiroketal system; however, 17-OH progesterone **6** was an inactive compound. Compound **18** (having two carbonyl groups) was more active than diosgenone, indicating this system’s importance regarding antiplasmodial activity.

SN-1 derivatives (**3**), **21**–**24** had a lower antiplasmodial effect than that of the natural compound (IC_50_ 21.4 µM) but, surprisingly, diacetate **23** (IC_50_ 17.3 µM) was more active in the FCB-2 strain (16.2 SI), as well as being less toxic.

The *Plasmodium berghei*-infected mice model was used as a biological model for *in vivo* antimalarial tests; more active, stable, less cytotoxic compounds **14** and **18** were selected to establish *in vivo* effects. Antimalarial activity was expressed as the percentage of parasite multiplication inhibition. This assay found that all mice, except one, had an average 0.4% infected erythrocytes 24 h post-infection, at which time treatment was started with 100 mg/kg weight of the derivative or 5 mg/kg weight of CQ. 

**Figure 2:**
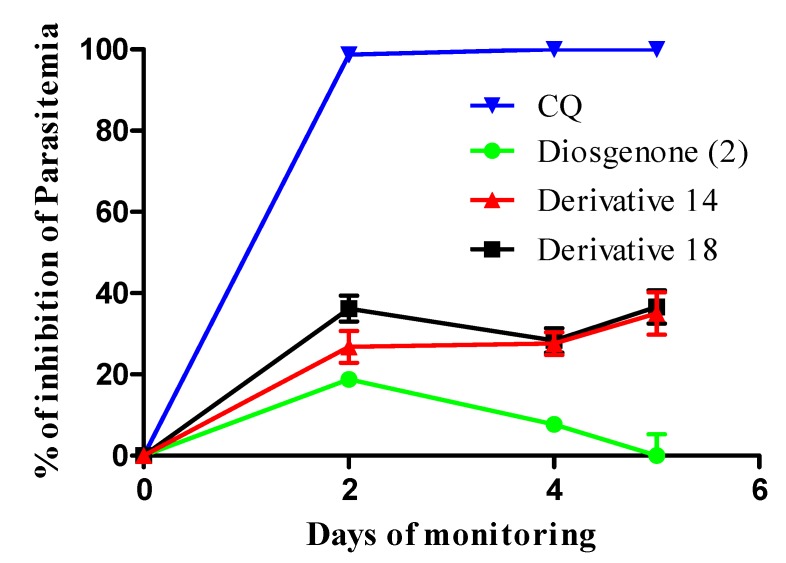
Percentage parasitemia inhibition in *P. berghei*-infected mice following treatment with derivatives **14** and **18**.

Regarding mortality, all mice were alive until termination of the experiment (day 5) when they were then anesthetized and sacrificed. CQ-treated mice had 100.0% reduction of parasitemia on the second day of treatment; by contrast, mice treated with diosgenone (**2**) showed no decrease in the amount of parasites, reaching 50.0% parasitaemias on the fifth day post treatment. Such parasitemia was higher than that found in treated mice or untreated controls with the vehicle, reaching rates of 36.0% parasitized erythrocytes. Treating mice with derivatives **14** and **18** caused a therapeutic effect, leading to 36.7% and 35.0% reduction in parasitemia, respectively (Figure 2). Their low solubility in water and organic solvents must be considered (given that this concentration was relatively high), since there would have been reduced gastrointestinal absorption which could have affected an optimal therapeutic dose. 

It would also be interesting to evaluate these drugs’ therapeutic effect using a chronic model of malaria (such as *Plasmodium chabaudi*). A striking finding emerged on the third day of treatment with both derivatives during the above experiments; many *P. berghei* sexual forms became reduced, along with asexual parasitaemia on the fourth day of treatment. It may be thought that *S. nudum* steroid derivatives act similarly to progesterone (α , β−unsaturated system) in inducing differentiation into gametocytes of asexual parasites. Lingnau *et al.* [19] treated *P. falciparum* cultures with different concentrations of insulin, progesterone, 17-β-estradiol and testosterone, finding that insulin asexual *in vitro* density increased compared to controls. By contrast, parasitemia did not increase when adding steroidal hormones progesterone, 17-β-estradiol and testosterone asexual, but gametocyte development was much more pronounced when using these steroids in the treatment, mainly progesterone.

### 2.3. Molecular Modeling

3D-QSAR analysis followed the methodology described in Section 4.3 of this manuscript. The best model obtained after PLS analysis was a mixture of 70% electrostatic field and 30% steric field. Cross-validated correlation coefficient q^2^ was 0.63, with 0.447 log units standard error of prediction at two components. This q^2^ value (greater than 0.5) indicated that a good predictive model had been obtained, in accordance with usual practice in the 3D-QSAR field. Compounds **4**, **11**, **21**, **22** and **25** behaved as statistical outliers; this was probably due to the conformational variability associated with the D-ring substituents’ flexible chains introducing additional noise to the model. These compounds also had low to moderate bio-activity so their contribution to the model would not have been expected to have been very significant.

The QSAR model was better depicted as 3D isosurfaces having standard deviation (stdev) multiplication values regarding the QSAR coefficient (coeff) at each point. This field gave information concerning the model’s salient features, specified by QSAR coefficients’ magnitude, scaled by associated variability at each grid point, specified by standard deviation [12].

**Figure 3:**
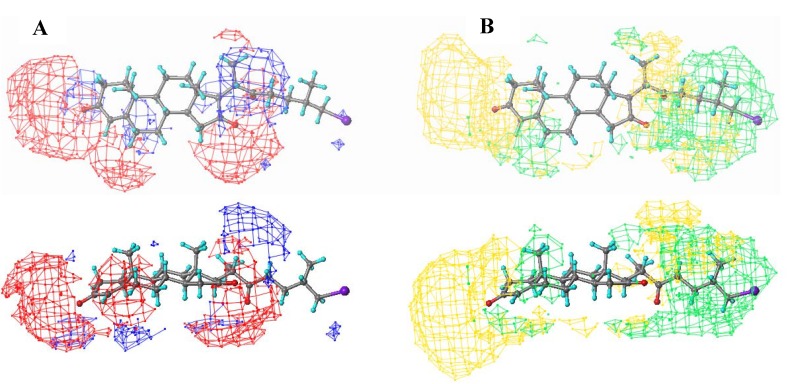
Electrostatic and steric contours mapped onto compound **15**.

Figure 3 presents a stdev*coeff field contoured on compound **15**; electrostatic and steric contributions are conveniently separated. Increased negative charge can be observed around steroidal skeleton A-, B- and D-rings (red mesh in Figure 3), associated with improved bio-activity, whereas increased positive charge around the D-ring decreased bio-activity.

**A**: Electrostatic field; **B**: Steric field. Electrostatic field - red and blue mesh surfaces; Steric field - yellow and green mesh surfaces; Two orientations are shown in the upper and lower parts of the Figure.

Regarding steric contribution, increased steric volume beyond the D-ring (green mesh) correlated with positive contribution to bioactivity, whereas the yellow mesh around the A-ring meant negative steric field contribution to bioactivity.

The fitting statistics for this model had a 0.958 correlation coefficient (estimated 0.1510 g units standard error). A comparison of the actual plot to the predicted one, showed the good correlation obtained. However, it should be mentioned that the standard error for the prediction given above only came from the statistical information provided by the model.

It could be assumed from such observations that the steroidal skeleton provided a rigid framework having the right shape to accommodate specific electrostatic interactions with charged residues at the receptor’s active site, leaving room for an additional increase in steric volume beyond the D-ring.

## 3. Experimental

### 3.1. Chemistry

#### 3.1.1. General Procedures

*S. nudum* steroid isolation and purification have already been described [5,6]; this involved reactions in an anhydrous nitrogen atmosphere using freshly-distilled, dry solvents prepared by standard methods. 4-Pregnene-3,20-dione, progesterone (**5**) and 17-hydroxy-4-pregnene-3,20-dione (**6**) were purchased from Sigma (St. Louis, MO, USA). Silica gel 60 (E. Merck, 70–230 mesh) was used for column chromatography purification. Thin-layer chromatography and nuclear magnetic resonance (NMR) were used for checking all new compounds’ purity. All reactions were monitored by TLC analysis on pre-coated silica gel F254 plates (E. Merck). Melting points (uncorrected) were determined by electrothermal equipment (Electrothermal 3100) and a spectrometer was used for infrared (IR) analysis (Thermo Nicolet Avatar 330 FT-IR, DTGS KBr). A spectrometer (Bruker AC 300) recorded ^1^H-, ^13^C- and ^1^H-decoupled spectra at 300 and 75 MHz. Chemical shifts (δ) obtained in CDCl_3_ were reported per million (ppm) relative to the residual solvent proton (7.26 ppm) and carbon signals (77.2). Only assignable signals were listed. Low resolution mass spectra were obtained in positive mode electrospray ionization mass spectra (ESI-MS) coupled to high-performance liquid chromatography (HPLC) apparatus (Agilent series 1200 model VL, Palo Alto, CA, USA) equipped with an atmospheric pressure ionization source.

#### 3.1.2. Chemical Transformations

The pertinent literature contains very few publications regarding diosgenone synthesis; these have dealt with the Oppenauer reaction with aluminum alkoxides [20], or ruthenium complexes [21]. However, yields have been poor or absent. Enzymatic transformations have also been attempted with structurally-similar molecules [22,23]. Obtaining diosgenone via Swern oxidation with subsequent double bond isomerization with oxalic acid in refluxing ethanol is reported here (84% overall yield).

*(25R)-4-Spirosten-3-one*, ***diosgenone*** (**2**). A mixture of oxalyl chloride (1.08 mmol, 136 mg, 2 eq.) in dichloromethane (DCM, 5 mL) was treated with dimethyl sulfoxide (DMSO, 0.23 mL, 1.08 mmol, 84 mg, 2 eq.) in DCM (5 mL) and stirred at −60 °C for 5 min. (25*R*)-5-spirosten-3β-ol (3β-hydroxy-5-spirostene, diosgenin **1**, 300 mg, 0.72 mmol, 1 eq.) in DCM (20 mL) was then added to the reaction mixture. Temperature was allowed to reach −10 °C after 20 min and then 0.65 mL *N,N*-diisopropyl ethylamine (3.73 mmol, 482 mg, 5.2 eq.) were added; stirring was continuous for 1 h to reach room temperature (RT) and HCl (20 mL, 5%) was added. The combined organic layer was washed with an aqueous solution (aq) of NaHCO_3_ (30 mL), dried (Na_2_SO_4_) and evaporated under reduced pressure. The residue was dissolved in an oxalic acid solution (1.4 g, 15.5 mmol, 21.5 eq.) in ethanol (20 mL) and heated to reflux for 5 h, extracted with DCM (3 × 15 mL), dried (Na_2_SO_4_) and evaporated under reduced pressure. The residue was purified by chromatography on silica and eluted with 4:1:1 hexane/DCM/Ethyl acetate (EtOAc), yielding diosgenone (**2**), as a white solid (255 mg, 84%), R*_f_* = 0.6 (hexane/EtOAc 4:1). Melting point (m.p.): 188–190 °C. IR (KBr): 2950, 1680, 1470. ^1^H- NMR (CDCl_3_) δ: 5.6 (s, 1H, H-4), 4.3 (dd, *J*_1_ = 14.0 Hz, *J*_2_ = 7.0 Hz, 1H, H-16), 3.4 (dd, *J*_1_ = 11.0 Hz, *J*_2_ = 3.0 Hz, 1H, H-26), 2.5 (dt, *J*_1_ = 17.0 Hz, *J*_2_ = 7.0 Hz 1H, H-2), 2.4 (dt, *J*_1_ = 15.0 Hz, *J*_2_ = 12.0 Hz, 1H, H-2), 1.12 (s, 3H, H-19), 0.88 (d, *J* = 7.0 Hz, 3H, H-21), 0.76 (s, 3H, H-18), 0.72 (d, *J* = 6.3, 3H, H-27); ^13^C-NMR (CDCl_3_) δ: 199.3 (C-3), 171.0 (C-5), 123.7 (C-4), 109.1 (C-22), 80.4 (C-16), 66.7 (C-26), 61.9 (C-17), 55.5 (C-14), 53.6 (C-9), 41.5 (C-20), 40.2 (C-13), 39.5 (C-12), 38.5 (C-10), 35.5 (C-1), 35.0 (C-8), 33.8 (C-2), 32.6 (C-6), 32.0 (C-7), 31.6 (C-15), 31.2 (C-23), 30.1 (C-25), 28.6 (C-24), 20.7 (C-11), 17.2 (C-19), 17.0 (C-27). 16.2 (C-18), 14.4 (C-21). MS (*m/z*) (%): 413.0 (M^+^, 100).

*(25R)-3-Methyl-spirostan-2,4-diene* (**7**). Diosgenone (**2**, 25 mg, 0.6 mmol, 1 eq.) in diethyl ether (5 mL) was treated with methyl magnesium bromide solution (0.44 mL, 0.62 mmol, 1.1 eq., 1.4 M toluene/THF 3:1) and stirred at −15 °C (one hour) followed by 2 h at RT. The reaction mixture was slowly treated with 5% aq NH_4_Cl (20 mL) and extracted with three × 20 mL portions of DCM. The combined organic extract was washed with water (20 mL) and brine (20 mL). The dried (Na_2_SO_4_) organic phase was concentrated under reduced pressure. The residue was purified by chromatography on a silica gel column (DCM/hexane 8:1) yielding compound **7** (14 mg, 54%): R*_f_* = 0.4 (DCM). M.p.: 172–174 °C. IR (KBr): 2928. ^1^H-NMR (CDCl_3_) δ: 5.8 (s, 1H, H-4), 5.4 (dd, *J* = 5.0 Hz, 1H, H-2), 4.4 (dt, *J*_1_ = 7.9 Hz, *J*_2_ = 1.8 Hz, 1H, H-16), 3.5 (d, *J* = 10.7 Hz, 2H, H-26), 1.7 (s, 3H, Me), 1.0 (s, 3H, H-19), 0.9 (d, *J* = 7.1 Hz, 3H, H-21), 0.8 (s, 3H, H-18), 0.72 (d, *J* = 6.3, 3H, H-27);^13^C-NMR (CDCl_3_) δ: 142.0 (C-5), 133.4 (C-3), 130.4 (C-2), 124.8 (C-4), 120.9 (C-6), 109.7 (C-22), 81.3 (C-16), 67.3 (C-26), 62.5 (C-17), 57.1 (C-14), 48.7 (C-9), 40.8 (C-20), 40.3 (C-13), 32.1 (C-6), 32.0 (C-15), 31.9 (C-23), 31.8 (C-25), 28.9 (C-24), 28.3 (C-11), 24.3 (Me), 21.3 (C-18), 19.4 (C-27), 16.8 (C-18), 14.9 (C-21). MS (*m/z*) (%): 411.2 (M^+^, 10). 

*(25R)-3-Benzyl-spirostan-4-en-3-ol* (**8**). Diosgenone (**2**, 50 mg, 0.12 mmol, 1 eq.) in diethyl ether (5 mL) was treated with benzylmagnesium bromide solution (0.14 mL, 0.14 mmol, 1.1 eq., 1.0 M THF) and treated as above. The residue was purified by chromatography on a silica gel column (DCM/hexane 8:1) yielding compound **8** as a white solid (56 mg, 92%): R*_f_* = 0.4 (hexane/EtOAc 8:1). M.p.: 203–205 °C. IR (KBr): 2930, 2880, 1600–1700. ^1^H-NMR (CDCl_3_) δ: 7.40–7.09 (m, 5H, H-Ar), 5.1 (s, 1H, H-4), 4.4 (dt, *J*_1_ = 7.9, *J*_2_ 1.7 Hz, 1H, H-16), 3.4 (d, *J* = 10.7 Hz, 2H, H-26), 2.8 (d, *J* = 14.7 Hz, 2H, Ar-CH_2_), 1.1 (s, 3H, H-19), 1.0 (d, 7.1 Hz, 3H, H-21), 0.8 (s, 3H, H-18), 0.8 (d, *J* = 6.2 Hz, 3H, H-27); ^13^C-NMR (CDCl_3_) δ: 146.8 (C-1'), 137.7 (C-5), 131.1 (C-4'), 129.3 (C-3', C-5'), 126.8 (C-2', C-4'), 126 (C-4), 109.7 (C-22), 81.2 (C-16), 71.7 (C-3), 67.3 (C-26), 62.5 (C-17), 56.4 (C-14), 54.8 (C-9), 47.2 (C-20), 40.3(C-13), 32.2 (C-6), 32.1 (C-15), 31.2 (C-22), 30.1 (C-25), 29.2 (C-26), 29.1 (C-10), 28.6 (C-23), 28.4 (C-24), 21.4 (C-19), 20.6 (C-11), 17.5 (C-27), 16.8 (C-18), 14.9 (C-21). MS (*m/z*) (%): 506.2 (M^+^ +1, 5), 487.2 (86).

*(25R)-3-p-Toluenehydrazine-spirostan-4-ene* (**9**). A mixture of diosgenone (**2**, 102 mg, 0.25 mmol, 1 eq.) and *p*TsNHNH_2_ (118 mg, 0.65 mmol, 2.6 eq.) in ethanol (5 mL) was heated to reflux for 5 h. The solvent was evaporated under vacuum and the residue was purified by chromatographic column on silica gel (hexane/EtOAc 5:1) yielding compound **9** as a white solid (122 mg, 84%): R*_f_* = 0.45 (hexane/EtOAc 5:1). M.p.: 148–150 °C. IR (KBr): 3508, 2930. ^1^H-NMR (CDCl_3_) δ: 7.9 (d, *J* = 8.1 Hz, 2H, H-Ar), 7.3 (d, *J* = 8.1 Hz, 2H, H-Ar), 5.8 (s, 1H, H-4), 4.4 (dt, *J*_1_ = 7.9 Hz, *J*_2_ = 1.8 Hz, 1H, H-16), 3.4 (d, *J* = 10.7 Hz, 2H, H-26), 2.4 (s, 3H, Ar-CH_3_), 1.0 (s, 3H, H-19), 0.9 (d, *J* = 7.2 Hz, 3H, H-21), 0.76 (s, 3H, H-18), 0.7 (d, *J* = 6.3 Hz, 3H, H-27); ^13^C-NMR (CDCl_3_) δ: 145.0 (C-5), 142.6 (C-3), 139.8 (C-1'), 136.4 (C-4'), 129.9 (C-3', C-5'), 129.1 (C-4), 128.5 (C-3', C-5'), 120.8 (C-2', C-6'), 109.7 (C-22), 81,6 (C-16), 62.5 (C-26), 56.2 (C-17), 53.9 (C-14), 42,3 (C-20), 35.7 (C-8), 29.2 (C-23), 29.0 (C-10), 28.8 (C-1), 28.7 (C-2), 28.5 (C-15), 28.4 (C-6), 28.2 (C-25), 28.1 (C-24), 20.8 (C-11), 20.6 (C-Me) 17.5 (C-19), 16.7 (C-27), 14.9 (C-21), 14.5 (C-18), 14.1 (C-21). MS (*m/z*) (%): 581.3 (M^+^, 5); 539.1 (36).

*(25R)-4,5-Epoxy-spirostan-3-one* (**10**). A mixture of diosgenone (**2**, 30 mg, 0.073 mmol, 1 eq.) and hydrogen peroxide (0.5 mL, 30%, 4.9 mmol, 67 eq.) in DCM/acetone 1:1 (20 mL) was treated with NaOH (1 mL, 2%) and stirred at RT for 48 h; then water was added to the reaction mixture and extracted with ethyl acetate (2 × 15 mL). The combined organic layer was washed with an aq NaHCO_3_ solution (30 mL), dried (Na_2_SO_4_) and evaporated under reduced pressure. The residue was purified by chromatography on a silica gel column (hexane/EtOAc 10:1) yielding compound **10** as a white solid corresponding to the mixture of epoxides α (70%) y β (30%) (21 mg, 65%): R*_f_* = 0.6 (hexane/EtOAc 9:1). M.p.: 184–186 °C. IR (KBr): 2899, 1715. ^1^H-NMR (CDCl_3_) δ: 4.4 (dt, *J*_1_ = 7.9 Hz, *J*_2_ = 1.8 Hz, 1H, H-16), 3.3 (d, *J* = 10.7 Hz, 2H, H-26), 3.0 (s, 1H, H-4β), 2.9 (s, 1H, H-4α), 1.2 (s, 3H, H-19), 1.0 (d, *J* = 7.1 Hz, 3H, H-21), 0.8 (s, 3H, H-18), 0.8 (d, *J* = 6.2 Hz, 3H, H-27); ^13^C-NMR (CDCl_3_) δ: 207.2 (C-3), 109.7 (C-22), 82.0 (C-16), 70.5 (C-5), 67.3 (C-26), 63.3 (C-4), 63.2 (C-17), 60.4 (C-17), 55.8 (C-14), 48.1 (C-9), 40.6 (C-12), 35.2 (C-20), 35.0 (C-17), 34.5 (C-8), 34.5 (C-10), 34.0 (C-2), 32.4 (C-1), 31.5 (C-15), 30.7 (C-6), 28.9 (C-23), 28.5 (C-7), 27.4 (C-24), 21.1 (C-11), 16.9 (C-27), 16.2 (C-19), 15.1 (C-21), 14.6 (C-18). MS (*m/z*) (%): 429.2 (M^+^, 82), 427.2 (12).

*(25R)-Spirostan-3-one* (**11**). A mixture of diosgenone (**2**, 40.8 mg, 0.1 mmol, 1 eq.) and palladium on carbon (Pd/C) 10% (2.0 mg, 10% mol, 0.01 mmol) in EtOAc (5 mL) was treated with hydrogen and stirred at RT overnight. The reaction mixture was filtered through a pad of celite and the solvent was evaporated under reduced pressure. The residue was purified by chromatography on a silica gel column (hexane/EtOAc 10:1) yielding compound **11** as a white solid (35 mg, 85%): R*_f_* = 0.4 (hexane/AcOEt 10:1). M.p.: 153–155 °C. IR (KBr): 2929, 1712. ^1^H-NMR (CDCl_3_) δ: 4.4 (dt, *J*_1_ = 7.9 Hz, *J*_2_ = 1.8 Hz, 1H, H-16), 3.4 (d, *J* = 10.7 Hz, 2H, H-26), 2.6 (d, *J* = 15.1 Hz, 2H, H-4), 1.1 (dt, *J*_1_ = 7.6 Hz, *J*_2_ 3.6 Hz, 2H, H-6), 1.0 (s 3H, H-18), 0.9 (d, *J* = 7.0 Hz, 3H, H-21), 0.7 (d, *J* = 6.3 Hz, 3H, H-27); ^13^C-NMR (CDCl_3_) δ: 210.0 (C-3), 109.7 (C-22), 81.2 (C-16), 67.3 (C-26), 62.6 (C-17), 56.7 (C-14), 44.6 (C-4), 41.1 (C-20), 40.3 (C-13), 38.9 (C-12), 38.2 (C-1), 38.1 (C-2), 35.8 (C-10), 35.0 (C-8), 31.6 (C-15), 30.7 (C-23), 28.6 (C-24, C-6), 20.8 (C-11), 19.9 (C-27), 17.5 (C-21), 16.8 (C-18), 14.9 (C-19). MS (*m/z*) (%): 415.2 (M^+^, 86), 397.1 (6).

*(25R)-1,4-Spirostadien-3-one* (**12**). A mixture of diosgenone (**2**, 50.0 mg, 0.1 mmol, 1 eq.), benzoic acid (14.8 mg, 0.12 mmol, 1.1 eq.) and 2,3-Dichloro-5,6-dicyano-p-benzoquinone (DDQ, 40.9 mg, 0.18 mmol, 1.8 eq.) was dissolved in 10.0 mL toluene and then heated to reflux for 12 h. The reaction mixture was filtered through celite and the solvent was evaporated under reduced pressure. The residue was purified by chromatography on a silica gel column (hexane/DCM/EtOAc 4:1:1) yielding compound **12** as a white solid (30 mg, 60%): R*_f_* = 0.3 (hexane/AcOEt 10:1). M.p.: 181–183 °C. IR (KBr): 2933, 2854, 1664, 1458. ^1^H-NMR (CDCl_3_) δ: 7.1 (d, *J* = 10.0, 1H, H-1), 6.3 (d, *J* = 10.0, 1H, H-2), 6.1 (s, 1H, H-4), 4.4 (dt, *J*_1_ = 7.9 Hz, *J*_2_ = 1.8 Hz, 1H, H-16), 3.6(d, *J* = 10.7 Hz, 2H, H-26), 2.6 (dt, *J*_1_ = 14.4, Hz *J*_2_ = 12.0 Hz, 2H, H-6), 1.3 (s, 3H, H-19), 0.9 (d, *J* = 7.1 Hz, 3H, H-21), 0.8 (d, *J* = 6.3 Hz, 3H, H-27), 0.7 (s, 3H, H-18); ^13^C-NMR (CDCl_3_) δ: 187.1 (C-3), 170.2 (C-5), 156.7 (C-1), 127.8 (C-2), 124.2 (C-4), 109.8 (C-22), 80.9 (C-16), 67.0 (C-26), 62.4 (C-17), 55.2 (C-14), 52.8 (C-9), 44.2 (C-10), 42.1 (C-20), 41.1 (C-13), 39.9 (C-12), 35.6 (C-8), 34.2 (C-7), 33.5 (C-6), 33.1 (C-15), 32.3 (C-23), 31.1 (C-25), 30.7 (C-24), 23.2 (C-11), 19.1 (C-19), 17.6 (C-27), 16.9 (C-18), 14.9 (C-21). MS (*m/z*) (%): 411.1 (M^+^, 100).

*(25R)-4-Spirosten-3-ol* (**13**). Diosgenone (2, 24 mg, 0.06 mmol, 1 eq.) in methanol (5 mL) was treated with NaBH_4_, (80 mg, 0.2 mmol, 3.3 eq.) and stirred at RT for 2 h overnight; water was then added to the reaction mixture and extracted with DCM (2 × 15 mL). The combined organic layer was washed with an aq NaHCO_3_ solution (30 mL), dried (Na_2_SO_4_) and evaporated under reduced pressure. The residue was purified by chromatography on a silica gel column (hexane/EtOAc 5:1) yielding compound 13 as a white solid (21 mg, 65%): R*_f_* = 0.4 (hexane/AcOEt 5:1). M.p.: 116–118 °C. IR (KBr): 3264, 2930, 2846, 1448. ^1^H-NMR (CDCl_3_) δ: 5.3 (d, *J* = 6.5 Hz, 1H, H-4), 4.4 (dt, *J*_1_ = 15.0, *J*_2_ = 7.5 Hz, 1H, H-16), 4.1 (dd, *J*_1_ = 18.0 Hz, *J*_2_ = 6.5 Hz, 1H, H-3), 3.5–3.3 (d, *J* = 10.7 Hz, 2H, H-26), 2.3 (dd, *J*_1_ = 14.0 Hz, *J*_2_ = 12.0 Hz, 1H, H-6), 1.0 (s, 3H, H-18), 0.9 (d, *J* = 6.9 Hz, 3H, H-21), 0.8 (s, 3H, H-19), 0.7 (d, *J* = 6.2 Hz, 3H, H-27); ^13^C-NMR (CDCl_3_) δ: 147.8 (C-5), 123.9 (C-4), 109.7 (C-22), 81.2 (C-16), 68.3 (C-3), 67.2 (C-26), 62.5 (C-17), 56.4 (C-14), 54.8 (C-9), 40.8 (C-20), 39.8 (C-13), 39.6 (C-12), 38.4 (C-10), 37.7 (C-1), 35.9 (C-8), 30.7 (C-25), 29.9 (C-2), 29.2 (C-24), 21.2 (C-11), 17.2 (C-19), 16.8 (C-27), 16.2 (C-18), 14.9 (C-21). MS (*m/z*) (%): 415.2 (M^+^, 80), 413.2 (21).

*(25R)-4,5-Epoxy-spirostan-16α-hydroxy-3-one* (**14**). A mixture of diosgenone (2, 200 mg, 0.48 mmol, 1 eq.) and NaHCO_3_ (1.89 g, 22.5 mmol, 47 eq.) in CHCl_3_-acetone-1.0 mM Na_2_EDTA (1:1:1, 37.5 mL, 4.65 g, 12.5 mmol, 26 eq.) was treated with oxone (6 g, 9.8 mmol, 20.4 eq.) in EDTA-Na_2_ (1 Mm) and stirred at RT for 48 h. The reaction mixture was extracted with DCM (2 × 15 mL) and the combined organic layer was washed with an aq NaHCO_3_ solution (30 mL), dried (Na_2_SO_4_) and evaporated under reduced pressure. The residue was purified by chromatography on a silica gel column (hexane/DCM /EtOAc 3:1:1) yielding compound 14 as a white solid corresponding to the mixture of epoxides α (70%) y β (30%) (170 mg, 80%): R*_f_* = 0.55 (hexane/AcOEt 3:1). M.p.: 145–147 °C. IR (KBr): 3450, 2940, 1710. ^1^H-NMR (CDCl_3_) δ: 3.6 (d, *J*_1_ = 10.7 Hz, *J*_2_ = 8.2 Hz, 2H, H-26), 3.0 (s, 1H, H-4α), 2.97 (s, 1H, H-4β), 1.8 (dd, *J*_1_ = 17.6 Hz, *J*_2_ = 2.4 Hz, 2H, H-2), 1.5 (d, *J* = 10.7 Hz, 2H, H-15), 1.0 (d, *J* = 7.1 Hz, 3H, H-21), 1.0 (s, 3H, H-18), 0.8 (s, 3H, H-19), 0.8 (d, *J* = 6.2 Hz, 3H, H-27). ^13^C-NMR (CDCl_3_) δ: 207.7 (C-3), 116.5 (C-22), 111.6 (C-16), 70.8 (C-5), 70.7 (C-3), 68.7 (C-26), 63.3 (C-4), 55.2 (C-17), 51.0 (C-15), 47.0 (C-9), 42.9 (C-15), 40.3 (C-12), 39.4 (C-20), 37.6 (C-8), 33.2 (C-1), 32.7 (C-6), 31.5 (C-23), 30.3 (C-25), 29.8 (C-24), 29.7 (C-1), 29.2 (C-7), 27.2 (C-2), 21.8 (C-11), 20.0 (C-11), 18.0 (C-27), 16.0 (C-19), 15.5 (C-18), 15.0 (C-21). MS (*m/z*) (%): 445.0 (M^+^, 3), 427.0 (96).

*(25R)-26-Iodine-cholest-4-en-3,16,22-trione* (**15**). A mixture of sodium iodide (NaI, 3 g, 20 mmol, 20 eq.), TMSCl (0.63 mL, 5 mmol, 5 eq.) and molecular sieve (5 g) in CH_3_CN (24 mL) was stirred at RT for 2 h. Compound 9 (446 mg, 1 mmol, 1 eq.) in DCM (15 mL) was added for 20 min. The reaction mixture was extracted with DCM (2 × 15 mL) and the combined organic layer was washed with an aq NaHCO_3_ solution (30 mL), dried (Na_2_SO_4_) and evaporated under reduced pressure. The residue was purified by chromatography on a silica gel column (hexane/DCM/EtOAc 2:1:1) yielding compound 15 as a white solid (404 mg, 75%): R*_f_* = 0.4 (hexane/EtOAc 2:1). M.p.: 148–150 °C. IR (KBr): 2942, 1733, 1717. ^1^H-NMR (CDCl_3_) δ: 5.7 (s, 1H, H-4), 3.3 (d, *J* = 4.9 Hz, 2H, H-26), 2.6 (d, *J* = 8.6 Hz, 1H, H-17), 1.6 (d, *J* = 16.4 Hz, 2H, H-23), 1.2 (d, *J* = 12.0 Hz, 3H, H-21), 1.1 (d, *J* = 6.2 Hz, 3H, H-27), 1.0 (s, 3H, H-18), 0.8 (s, 3H, H-19); ^13^C-NMR (CDCl_3_) δ: 218.2 (C-16), 213.7 (C-22), 199.9 (C-3), 170.8 (C-5), 124.9 (C-4), 66.9 (C-17), 53.9 (C-9), 51.2 (C-14), 44.0 (C-), 42.5 (C-13), 40.2 (C-20), 39.3 (C-12), 39.2 (C-15), 39.0 (C-10), 36.2 (C-23), 36.1 (C-8), 34.6 (C-1), 34.5 (C-24), 33.2 (C-2), 32.7 (C-6), 30.6 (C-7), 21.2 (C-25), 20.9 (C-27), 18.6 (C-11), 18.1 (C-26), 17.6 (C-19), 16.1 (C-21), 13.8 (C-18). MS (*m/z*) (%): 539.1 (M^+^, 96), 500.1 (5).

*(25R)-3-Acetoxy-spirostan-5-ene* (**16**). A mixture of diosgenine (**1**, 500 mg, 1.2 mmol, 1 eq.), acetic anhydride (1 mL, 1.05 mmol, 8.8 eq.) and ZnCl_2_ (0.5 g, 3.6 mmol, 3 eq.) was stirred at RT for 3 h. An Na_2_CO_3_ solution was added to the reaction mixture and extracted with EtOAc (2 × 15 mL), dried (Na_2_SO_4_) and evaporated under reduced pressure. The residue was purified by chromatography on a silica gel column (hexane/EtOAc/DCM 5:1:1) yielding compound **16** as a white solid (452 mg, 82%) R*_f_* = 0.5 (hexane/EtOAc 3:1). M.p.: 182–184 °C. ^1^H-NMR (CDCl_3_) δ: 5.4 (d, 1H, *J* = 4.7 Hz, H-6), 4.6 (m, 1H, H-3), 4.4 (dt, 1H, *J*_1_ = 15 Hz, *J_2_* = 7 Hz, H-16), 3.5(d, *J* = 10.7 Hz, 2H, H-26), 2.1 (s, 3H, 3-OCOCH_3_), 1.6 (s 3H, H-18), 1.0 (s 3H, H-19), 0.9 (d, 3H, *J* = 7.1 Hz, H-21), 0.8 (d, 3H, *J* = 6.3 Hz, H-27); ^13^C-NMR (CDCl_3_) δ: 170.5 (3-OCOCH_3_), 139.6 (C-5), 122.3 (C-6), 109.2 (C-22), 80.8 (C-16), 73.8 (C-3), 66.8 (C-26), 62.0 (C-17), 56.4 (C-14), 49.9 (C-9), 41.6 (C-20), 40.2 (C-13), 39.7 (C-4), 38.0 (C-12), 36.9 (C-1), 36.7 (C-10), 32.0 (C-7), 31.8 (C-15), 31.4 (C-8), 31.3 (C-23), 30.3 (C-25), 28.8 (C-2), 27.7 (C-24), 21.4 (3-OCH_3_), 20.8 (C-11), 19.3 (C-19), 17.1 (C-27), 16.2 (C-18), 14.5 (C-21).

*(25R)-5,6-Epoxy-spirostan-3-ol* (**17**). A mixture of diosgenine (**1**, 100 mg, 2.0 mmol, 1 eq.) and NaHCO_3_ (1.89 g, 22.5 mmol, 11 eq.) in CHCl_3_-acetone-1.0 mM Na_2_EDTA (1:1:1, 37.5 mL) was treated with oxone (6.0 g, 9.8 mmol) in Na_2_EDTA 1mM and stirred at RT for 16 h. The reaction mixture was extracted with DCM (2 × 15 mL). The combined organic layer was washed with an aq NaHCO_3_ solution (30 mL), dried (Na_2_SO_4_) and evaporated under reduced pressure. The residue was purified by chromatography on a silica gel column (hexane/DCM/EtOAc 1:1:1) yielding compound **17** as a white solid corresponding to the mixture of epoxides α (70%) y β (30%) (85.1 mg, 70.1%): R*_f_* = 0.4 (hexane/EtOAc 2:3). M.p.: 97–99 °C. ^1^H-NMR (CDCl_3_) δ: 4.38 (dd, 1 H, *J =* 7.4 Hz, H-16), 3.9 (m, 1H, H-3), 3.5 (dd, 1H, *J* = 11, 3 Hz, H-26), 3.36 (dd, 1H, *J* = 11 Hz, H-26), 3.10 (d, 1H, *J* = 4Hz, H-6β), 2.92 (d, 1H, *J* = 4 Hz, H-6α), 1.09 (s, 3H, H-19), 0.97 (d, 3H, *J* = 7 Hz, H-21), 0.80 (d,3H, *J* = 6 Hz, H-27), 0.74 (s, 3H, H-18); ^13^C-NMR (CDCl_3_) δ: 110.0 (C-22), 81.3 (C-16), 69.3 (C-3), 67.5 (C-26), 66.4 (C-5), 62.6 (C-17), 59.8 (C-6), 55.7 (C-14), 43.3 (C-4), 42.3 (C-12), 41.0 (C-13), 40.5 (C-20), 35.7 (C-10), 32.4 (C-1), 31.8 (C-15), 31.7 (C-7), 31.0 (C-23), 30.1 (C-9), 30.0 (C-26), 29.7 (C-2), 29.5 (C-24), 22.5 (C-11), 17.0 (C-27), 16.9 (C-19), 16.7 (C-21), 15.2 (C-18).

*(25R)-4-Spirosten-3,6-dione* (**18**). A mixture of diosgenine (**1**, 50 mg, 0.12 mmol, 1 eq.) and pyridinium chlorochromate (PCC, 100 mg, 0.46 mmol, 3.8 eq.) in DCM (30 mL) was stirred at RT for 5 h. The reaction mixture was extracted with DCM (2 × 15 mL). The combined organic layer was washed with an aq NaHCO_3_ solution (30 mL), dried (Na_2_SO_4_) and evaporated under reduced pressure. The residue was purified by chromatography on a silica gel column (hexane/DCM/EtOAc 2:1:1) yielding compound **18** as a yellow solid (28 mg, 55%): R*_f_* = 0.3 (hexane/AcOEt/DCM 7:1:1). M.p.: 200–202 °C. IR (KBr): 2951, 2925, 2850, 1700. ^1^H-NMR (CDCl_3_) δ: 6.2 (s, 1H, H-4), 4.42 (dd, *J*_1_ = 7.6 Hz, *J*_2_ = 13.8 Hz, 1H, H-16), 3.5(d, *J* = 10.7 Hz, 2H, H-26), 2.8 (d, *J* = 14.7 Hz, 2H, H-7), 1.2 (s, 3H, H-19), 1.0 (d, *J* = 7.1 Hz, 3H, H-21), 0.8 (d, *J* = 6.2 Hz, 3H, H-27), 0.7 (s, 3H, H-18); ^13^C-NMR (CDCl_3_) δ: 202.1 (C-7), 199.6 (C-3), 160.8 (C-5), 125.9 (C-4), 109.6 (C-22), 80.5 (C-16), 67.2 (C-26), 62.1 (C-17), 56.5 (C-14), 51.1 (C-9), 47.0 (C-7), 41.9 (C-20), 40.7 (C-12), 39.9 (C-13), 39.4 (C-10), 35.7 (C-1), 34.2 (C-2), 33.9 (C-8), 31.7 (C-15), 31.5 (C-23), 30.4 (C-25), 29.0 (C-24), 20.9 (C-11), 17.8 (C-19), 17.3 (C-27), 16.5 (C-18), 14.7 (C-21). MS (*m/z*) (%): 427.0 (M^+^, 100).

*(25R)-3,5-Spirostadiene* (**19**). HCl (2.5 mL, 36.5%, 31.8 mmol, 635 eq.) was added to a solution of diosgenine (**1**, 20 mg, 0.05 mmol, 1 eq.), zinc powder (250 mg, 3.8 mmol, 76 eq.) in ethanol (5 mL) and stirred at RT for 2 h. NaOH (15 mL, 10%) was added to the reaction mixture and extracted with DCM (2 × 15 mL). The combined organic layer was washed with an aq NaHCO_3_ solution (30 mL), dried (Na_2_SO_4_) and evaporated under reduced pressure. The residue was purified by chromatography on a silica gel column (DCM/methanol (MeOH) 100:1) generating **19** as white solid (13 mg, 71%): R*_f_* = 0.5 (DCM/MeOH 20:1). M.p.: 116–118 °C. ^1^H-NMR (CDCl_3_) δ 5.9: (d, *J* = 4.0 Hz, 1H, H-3), 5.8 (d, *J* = 4.0 Hz, 1H, H-4), 5.3 (dd, *J* = 6.0, 1.3 Hz, 1H, H-6, 4.39 (dt, *J*_1_ = 7.6 Hz, *J*_2_ = 13.8 Hz, 1H, H-16), 3.4 (d, *J* = 10.7 Hz, 2H, H-26), 1.2 (s, 3H, H-19), 1.0 (d *J* = 7.1 Hz, 3H, H-21), 0.9 (d, *J* = 6.2 Hz, 3H, H-27), 0.8 (s, 3H, H-18); ^13^C-NMR (CDCl_3_) δ: 141.9 (C-5), 129.3 (C-3), 125.4 (C-4), 123.1 (C-6), 109.7 (C-22), 81.2 (C-16), 67.2 (C-26), 62.5 (C-17), 57.2 (C-14), 48.7 (C-9), 41.8 (C-20), 40.3 (C-12), 35.3 (C-10), 34.6 (C-1), 34.0 (C-8), 33.7 (C-15), 33.4 (C-23), 32.0 (C-8), 30.6 (C-25), 28.9 (C-24), 23.3 (C-2), 21.1 (C-11), 19.1 (C-19), 18.2 (C-27), 17.5 (C-18), 16.6 (C-21).

*(25R)-23-Acetyl-3,16-diacetoxy-22,23-pyran-cholesta-5,22-diene* (**20**). ZnCl_2_ (1.0 g, 7.2 mmol, 6 eq.) was added to a solution of diosgenine (**1**, 500 mg, 1.2 mmol, 1 eq.) in acetic anhydride (5 mL, 0.5 mmol, 0.4 eq.) and stirred at RT for 72 h. Water was added to the reaction mixture and extracted with EtOAc (3 × 15 mL). The combined organic layer was washed with an aq NaHCO_3_ solution (30 mL), dried (Na_2_SO_4_) and evaporated under reduced pressure. The residue was purified by chromatography on a silica gel column (hexane/EtOAc/DCM, 9:1:1) yielding compound **20** as a white solid (620 mg, 96%): R*_f_* = 0.6 (hexane/EtOAc 4:1).^1^H-NMR (CDCl_3_) δ: 5.3 (d, *J* = 4.3Hz, 1H, H-6), 5.1 (dt, *J*_1_ = 5.9 Hz, *J*_2_ = 4.9 Hz, 1H, H-16), 4.6 (dt, *J* = 12.9, 8.1 Hz, 1H, H-3), 4.0 (dd, *J*_1_ = 10.7 Hz, *J*_2_ = 4.7 Hz, 2H, H-26), 2.9 (dd, *J* = 6.5 Hz, 1H, H-20,), 2.2 (s, 3H, 28-COCH_3_), 2.0 (s, 3H, 3-OCOCH_3_), 1.9 (s, 3H, 16-OCOCH_3_), 1.2 (d, *J* = 7.0 Hz, 3H, H-21,), 1.0 (s, 3H, H-19), 0.9 (s, 3H, H-18,), 0,8 (d, *J* = 6.3, 3H, H-27); ^13^C-NMR (CDCl_3_) δ: 198.1 (C-27), 171.3 (C-22), 170.6 (16-OCOCH_3_), 170.4 (3-OCOCH_3_), 139.7 (C-5), 122.2 (C-6), 106.9 (C-23), 75.1 (C-16), 73.8 (C-3), 71.5 (C-26), 55.9 (C-17), 54.2 (C-14), 49.9 (C-9), 42.2 (C-13), 39.7 (C-12), 38.0 (C-4), 36.8 (C-1), 36.5 (C-10), 34.8 (C-15), 32.8 (C-20), 31.6 (C-24), 31.5 (C-7), 31.3 (C-8), 29.7 (COCH_3_), 28.4 (C-28), 27.8 (C-2), 22.2 (C-11), 21.2 (C-25), 21.3 (COCH_3_), 20.7 (C-19 C24), 19.3 (C-21), 19.2 (C-18). 

*(25R)-16-Acetoxy-4-cholesten-3,22-dione-26-al* (**21**). DMSO (0.2 mL, 2.8 mmol, 93 eq.) was added dropwise to a solution of (COCl)_2_ (0.1 mL, 2.3 mmol, 29 eq.) in DMC (5 mL) at −60 °C and the resulting solution was stirred for 15 min. A solution of SN-1 (14.2 mg, 0.03 mmol, 1 eq.) in DMC (3 mL) was added dropwise to this solution and stirred for 30 min. (*i*Pr)_2_NEt (0.62 g, 4.8 mmol, 162 eq.) was added dropwise, and the solution was warmed at RT for 1 h. The solution was diluted with water (10 mL) and extracted with DCM (4 × 15 mL). The organic extracts were combined, washed with water (30 mL), brine (30 mL), dried over Na_2_SO_4_, and concentrated *in vacuo* to yield **21** as a white solid. (8.2 mg, 58.1%): R*_f_* = 0.6 (hexane/EtOAc 1:2).^ 1^H-NMR (CDCl_3_) δ: 9.6 (s, 1H, CHO), 5.7 (s, 1H, H-4), 4.9 (dt, *J* = 10.7, 5.9 Hz, 1H, H-16), 2.6 (dd, *J* = 16.4, 86 Hz, 2H, H-23), 2.0 (s, 3H, 16-OCOCH_3_), 1.2 (s, 3H, H-19), 1.1 (d, *J* = 7.1 Hz, 3H, H-27), 1.0 (d, *J* = 7.1 Hz, 3H, H-21), 0.9 (s, 3H, H-18); ^13^C-NMR (CDCl_3_) δ: 212.2 (C-22), 204.4 (C-26), 199.4 (C-3), 170.7 (16-OCOCH_3_), 170.6 (C-5), 124.0 (C-4), 78.4 (C-16), 57.7 (C-17), 53.4 (C-9), 52.8 (C-14), 47.9 (C-25), 45.6 (C-20), 43.6 (C-13), 39.3 (C-10), 38.7 (C-12), 38.5 (C-23), 35.6 (C-15), 34.9 (C-8), 34.1 (C-1), 33.9 (C-2), 32.6 (C-6), 31.7 (C-7), 23.9 (C-24), 21.2 (16-OCOCH_3_), 20.6 (C-11), 17.3 (C-19), 16.3 (C-18), 13.6 (C-21), 13.3 (C-27).

*(25R)-16-Acetoxy-26-hydroxy-cholestan-3,22-dione* (**22**)*.* A mixture of SN-1 (20.9 mg, 0.04 mmol, 1 eq.) and Pd/C 10% (1.0 mg, 10% mol, 0.1 eq.) in EtOAc (5 mL) was treated with hydrogen and stirred at RT overnight. The reaction mixture was filtered through celite and the solvent was evaporated under reduced pressure. The residue was purified by chromatography on a silica gel column (hexane/EtOAc 10:1) yielding compound **22** as a white solid (18.1 mg, 96.1%): R*_f_* = 0.4 (hexane/AcOEt 2:1). ^1^H-NMR (CDCl_3_) δ: 4.83 (dd, *J*_1_ =10.7, *J*_2_ = 5.9 Hz, 1H, H-16), 3.33 (d, , 2H, H-26), 2.4 (dd, , 2H, H-24), 2.0 (s, 3H, 28-COCH_3_), 1.1 (d, *J* = 7.1 Hz, 3H, H-21), 1.0 (s, 3H, H-19), 0.9 (s, 3H, H-18), 0.8 (d, *J* = 6.4 Hz, 3H, H-27); ^13^C-NMR (CDCl_3_) δ: 212.8 (C-22), 212.1 (C-3), 170.0 (16-OCOCH_3_), 77.4 (C-16), 65.7 (C-26), 56.7 (C-17), 52.2 (C-14), 51.9 (C-9), 51.7 (C-5), 46.4 (C-20), 42.6 (C-4), 42.5 (C-13), 38.4 (C-1), 38.2 (C-12), 38.1 (C-23), 38.0 (C-2), 37.0 (C-15), 36.7 (C-10), 33.8 (C-25), 33.5 (C-8), 30.1 (C-6), 29.6 (C-7), 25.0 (C-24), 21.2 (16-OCOCH_3_), 19.8 (C-19), 19.6 (C-18), 19.4 (C-11), 15.9 (C-27), 15.1 (C-21).

*(25R)-16,26-Diacetoxy-4-cholesten-3,22-dione* (**23**). Acetyl chloride (0.15 mL, 1.8 mmol, 450 eq.) was added to a mixture of SN-1 (20.1 mg, 0.04 mmol, 1 eq.) in pyridine (2 mL) and the resulting solution was stirred for 30 min at RT. Water was added to the reaction mixture and extracted with EtOAc (3 × 15 mL). The combined organic layer was washed with an aq NaHCO_3_ solution (30 mL), dried (Na_2_SO_4_) and evaporated under reduced pressure. The residue was purified by chromatography on a silica gel column (hexane/EtOAc, 5:2) yielding compound **23** as a white solid (22.1 mg, 100%): R*_f_* = 0.6 (hexane/EtOAc 4:1). ^1^H-NMR (CDCl_3_) δ: 5.68 (s, 1H, H-4), 4.92 (dd, *J*_1_ = 10.7 Hz, *J*_2_ = 5.9 Hz, 1H, H-16), 3.55 (d, *J* = 10.7 Hz, 2H, H-26), 2.4 (dd, *J* = 16.4 Hz, 2H, H-23), 2.0 (s, 6H, COCH_3_), 1.2 (s, 3H, H-19), 1.1 (d, *J* = 7.1 Hz, 3H, H-21), 0.9 (s, 3H, H-18), 0.8 (d, *J* = 6.3 Hz, 3H, H-27). ^13^C-NMR (CDCl_3_) δ: 212.8 (C-22), 199.4 (C-3), 171.2 (24-OCOCH3), 170.6 (16-OCOCH3), 124.0 (C-4), 78.6 (C-16), 68.9 (C-26), 57.8 (C-17), 53.5 (C-14), 53.4 (C-9), 52.8 (C-5), 47.9 (C-20), 43.6 (C-4), 39.3 (C-13), 39.1 (C-1), 38.5 (C-12), 35.6 (C-23), 34.9 (C-2), 34.1 (C-15), 33.9 (C-10), 32.6 (C-25), 32.1 (C-8), 31.7 (C-6), 26.7 (C-7), 21.2 (C-24), 21.2 (16-OCOCH_3_), 20.8 (24-OCOCH_3_), 17.8 (C-19), 15.9 (C-27), 15.1 (C-21), 13.1 (C-18).

*(25R)-16-Acetoxy-4-cholesten-3,22,26-triol* (**24**). SN-1 (19.1 mg, 0.04 mmol, 1 eq.) in MeOH (5 mL) was treated with NaBH_4_ (38.2 mg, 1 mmol, 25 eq.) and stirred at −15 °C for 2 h and then at RT overnight. Water was added to the reaction mixture and extracted with DCM (2 × 15 mL). The combined organic layer was washed with an aq NaHCO_3_ solution (30 mL), dried (Na_2_SO_4_) and evaporated under reduced pressure. The residue was purified by chromatography on a silica gel column (hexane/EtOAc 1:1) yielding compound 24 as a white solid (12 mg, 63%): R_f_ = 0.3 (hexane/AcOEt 1:1). M.p.: 144–146 °C. ^1^H-NMR (CDCl_3_) δ: 5.3 (d, *J* = 6.7 Hz, 1H, H-4), 5.0 (dt, *J*_1_ = 10.7 Hz, *J*_2_ = 5.9 Hz, 1H, H-16), 4.1 (q, *J* = 6.7 Hz, 1H, H-3,), 3.5 (tq, *J* = 8.0, 7.0 Hz, 2H, H-22), 3.4 (d, *J* = 10.4 Hz, 2H, H-26), 2.0 (s, 3H, 16-OCOCH_3_), 1.2 (d, *J* = 7.1 Hz, 2H, H-21), 1.0 (s, 3H, H-19), 0.8 (s, 3H, H-18); 0.7 (d, *J* = 7.1 Hz, 3H, H-27); ^13^C-NMR (CDCl_3_) δ: 170.7 (16-OCOCH_3_), 147.1 (C-5), 123.6 (C-4), 79.9 (C-16), 73.3 (C-22), 68.1 (C-26), 67.9 (C-3), 58.4 (C-17), 54.1 (C-9), 53.4 (C-14), 43.3 (C-13), 39.9 (C-12), 38.4 (C-20), 37.2 (C-10), 35.9 (C-25), 35.3 (C-8), 35.2 (C-15), 34.3 (C-1), 32.8 (C-6), 32.0 (C-7), 31.6 (C-24), 29.9 (C-23), 29.4 (C-2), 21.4 (C-18), 21.0 (16-OCOCH_3_), 20.7 (C-11), 18.8 (C-19), 16.6 (C-27), 14.2 (C-21).

*(25R)-16-Hydroxy-4-cholesten-3,22-dione-26-al* (**25**). DMSO (0.2 mL, 2.8 mmol, 93 eq.) was added dropwise to a solution of (COCl)_2_ (0.1 mL, 2.4 mmol, 47 eq.) in DCM (5 mL) at −60 °C and the resulting solution was stirred for 15 min. A solution of SN-2 (21.5 mg, 0.05 mmol, 1 eq.) in DMC (3 mL) was added dropwise to this solution and stirred for 30 min. (*i*Pr)_2_NEt (0.62 g, 4.8 mmol, 162 eq.) was added dropwise, and the solution was warmed at RT for 1 h. The solution was diluted with water (10 mL) and extracted with DMC (4 × 15 mL). The organic extracts were combined, washed with water (30 mL), brine (30 mL), dried over Na_2_SO_4_, and concentrated in vacuo to yield compound **25** as a white solid (14.9 mg, 69.8%): R*_f_* = 0.5 (hexane/EtOAc 1:2). ^1^H-NMR (CDCl_3_) δ: 9.6 (s, 1H, CHO), 5.7 (s, 1H, H4), 2.9 (s, 1H, H-17), 2.6 (dd, *J* = 16.4 Hz, 2H, H-23), 1.1 (d, *J* = 7.1 Hz, 3H, H-21), 1.0 (d, *J* = 6.5 Hz, 3H, H-27), 0.8 (s, 3H, H-18); ^13^C-NMR (CDCl_3_) δ: 216.8 (C-16), 213.0 (C-22), 204.4 (C-26), 199.7 (C-3), 169.9 (C-5), 124.2 (C-4), 66.3 (C-17), 53.2 (C-9), 50.5 (C-14), 45.6 (C-25), 43.2 (C-20), 41.7 (C-13), 39.4 (C-15), 38.9 (C-10), 38.4 (C-12), 37.1 (C-23), 35.4 (C-1), 34.6 (C-8), 33.9 (C-2), 32.5 (C-6), 31.9 (C-7), 24.0 (C-24), 20.5 (C-11), 17.3 (C-19), 15.4 (C-18), 14.1 (C-21), 13.1 (C-27).

### 3.2. Biological Assays

#### 3.2.1. *In Vitro* Antiplasmodial Activity

A 10 mg/mL stock solution was prepared. 2.0 mg of derivatives having greater than 95% purity (as observed by TLC and ^1^H-NMR spectra) were dissolved in 200 μL pure DMSO. A 50 μL sample was extracted from this solution and adjusted to 1,000 μL with RPMI-1640 without hypoxanthine until a final 500 μg/mL concentration was reached. The *P. falciparum* FcB-2 (Colombia) and NF54 (The Netherlands) strains were used for assessing anti-malarial activity.

Trager and Jensen’s method was used for culturing strains [24,25]. The methods described by Desjardins *et al.* [26] were used for evaluating the structural analogs’ *in vitro* antiplasmodial activity, with some modifications. 200 μL parasitized erythrocytes (1.0% parasitemia, 1.8% hematocrit) were placed in 96-well plates preloaded with seven concentrations (100 µg/mL–3.12 µg/mL) of each derivative in triplicate or with serial dilutions of chloroquine in positive control wells (3,753 nM up to 59 nM). 1.0 µCi/mL of [3H] hypoxanthine (MP Biomedicals, Santa Ana, CA, USA) was added to each well and the plates were then incubated for 48 h at 37 °C in 5% CO_2_, 5% O_2_ in a balanced N_2_ atmosphere. Parasite DNA was harvested and radioactivity was determined by automatic TDCR liquid scintillation counter plate (Chameleon, Hidex Oy, Finland). Experiments were repeated twice with two or three replicates each. A nonlinear regression sigmoidal dose-response (variable slope) model was used to estimate concentration inhibiting 50% growth (IC_50_) values for each derivative.

#### 3.2.2. Cytotoxicity

HepG2-A16 human hepatoma cells (ATCC Hb-8065) were maintained in DMEM-F12 (Gibco, Carlsbad, CA, USA) supplemented with 10% bovine fetal serum (BFS) (Gibco) and 150 μg/mL penicillin/streptomycin (Sigma) at 37 °C in a 5% CO_2_ humidified atmosphere [27].

Steroidal derivatives’ cytotoxicity regarding the HepG2-A16 cell line was evaluated by colorimetric test using 3-(4,5-dimethylthiazolyl-2)-2,5-diphenyltetrazolium bromide (MTT), as described by Mosmann [28]. In brief, the cells were cultured in a 96-well flat-bottomed plate (2 × 10^5^ cells/well in 100 μL complete medium) and incubated for 24 h at 37 °C in a 5% CO_2_ humidified atmosphere to allow monolayer formation. An aliquot of each derivative dilution (100 μL, 1,000, 100, 10 and 1 μg/mL) was then added to the wells in triplicate. The plates were incubated for another 48 h and then 30 μL MTT (2 mg/mL) was added and the plates were incubated again for 4 h; DMSO (96%, 130 μL) was added and plates incubated for a further 20 min at RT. The DMSO diluent agent was evaluated in complete medium and negative controls (untreated cells) were included. Absorbance was measured at 550 nm and statistical Prism software version 5.0 (GraphPad Software Inc., La Jolla, CA, USA) was used for calculating toxic concentration (CC_50_). Amphotericin B was used as positive control in this assay.

#### 3.2.3. The *in Vivo* Effects of Derivatives **14** and **18**

Antimalarial activity was assessed *in vivo* in the *P. berghei* rodent malaria model according to Peters’ 4-day suppressive test in *P. berghei*-infected (ANKA strain), 5–6 week-old Balb/C mice (males and females) weighing 23 ± 5 g; parasitemia kinetics were evaluated following treatment [2]. The mice were managed according to Colombian animal protection regulations (statute 84/1989), after approval had been obtained from the Universidad de Antioquia’s Research Center’s (SIU) Ethics Committee. Assays were carried out in the SIU animal housing’s testing room, coordinated by the Tropical Diseases Program (PECET). The donor mice were anesthetized with a 5 mg/kg xylazine and 50 mg/kg ketamine cocktail; blood was then collected via intracardiac route and 100 uL of 5 × 10^6^ *P. berghei* ANKA-parasitised parasitized erythrocytes per experimental mouse were inoculated. Mouse infection was checked one day later by thin smear blood test and Giemsa staining. Batches of 5 animals per group were then randomly distributed after infection and each group of mice received 100 μL of their respective treatment via oral-gastric tube (day 0) [*i.e.*, 100 mg/kg compound **2**, derivative **14**, **18**, negative control (standard suspending vehicle – SSV] or 5 mg/kg chloroquine in positive control. Mice were treated again with the same dose given on day 0 after 24, 48 and 72 h. Each mouse had a thin smear blood test on days 2, 4 and 5 (48, 96 and 120 h post-infection) and parasitemia was counted in 10,000 red blood cells (results expressed as percentage parasitized RBC). The difference between control group mean value (taken as 100%) and those of the experimental groups was also calculated and expressed as percent reduction using the following equation:

*Inhibition* = 100 − (mean percentage parasitemia treated/mean percentage parasitemia control with SSV) × 100)

### 3.3. Computational Methods

Each compound’s geometry was optimized by B3LYP/6-31G(d) [29], using the Gaussian 03 software [30]. Electrostatic charges were calculated by fitting electrostatic potential to nuclear positions, according to the CHELPG scheme [31].

#### 3.3.1. CoMFA Details

Steric and electrostatic 3D fields were calculated by the CoMFA method using the interaction between each catalyst and a probe atom. The probe atom had a specific charge and steric properties for evaluating interaction energy at each point on a grid. The probe atom selected was a Sp^3^ C atom having a + 1 point charge (*i.e.*, Tripos C(3) force field atom) [32]; it was used to calculate Van der Waals (steric) interactions. 3D-QSAR analysis was carried out using the CoMFA module [33] and Sybyl 8.0 software [34].

#### 3.3.2. Alignment Rule

All structures had to be aligned in a common framework when using 3D-QSAR for comparing the 26 compounds tested here [35]; the C atoms shared between fused A-B, B-C and C-D rings were selected. The best grid spacing results were obtained with a 1.0 Å lattice spacing. Such late value was considered an optimum value since higher precision in evaluating the 3D field by introducing a finer grid would have resulted in an increase of so-called “brown noise” caused by the grid size sensitivity of the statistical technique used for generating the models [36].

#### 3.3.3. PLS Analysis

Partial least squares (PLS) analysis was performed for different combinations of field descriptors. Autoscaling was used for PLS calculations regarding combined fields where each field was scaled to give unit value variance. Software calculated each field’s standard deviation (SD) and divided each value by the corresponding SD to assign the same prior importance to each variable in the analysis. Leave one out (LOO) [37] cross-validated PLS analysis was initially used for determining statistical model robustness and the optimal number of components. This involved examining the predictive residual sum of squares (PRESS) and using the cross-validated regression coefficient (q^2^) as guidelines. q^2^ was defined as:

PRESS =
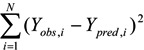
(1)

q^2^ = 1 − PRESS/SSD (2)

where *Y_obs,I_* and *Y_pred,I_* were actual and predicted dependent variables (respectively) and SSD the sum of each dependent variable’s squared deviation from the mean of all dependent variables. Some authors [38] have estimated that a greater than 0.3 q^2^ value has a 95% confidence limit. Usual drug design practice considers a model having a q^2^ greater than 0.5 to be valid, *i.e.*, halfway between perfect predictions (q^2^ = 1.0) and no prediction at all (q^2^ = 0.0). The optimum number of components was determined by minimizing PRESS while maximizing q^2^ values; the model having fewer components was selected whenever an increase in q^2^ with an additional component was less than 5%. Adding more components improved the fitting statistics but had two disadvantages: it complicated the model and made it lose its predictive ability. A final model was obtained by non-cross-validated PLS analysis for ascertaining the optimum number of components. The experimental activity values were transformed to a logarithmic scale to improve data distribution, as recommended by usual QSAR practice [12]; the transformation used was thus Log (1/IC_50_).

## 4. Conclusions

Only a few reports concerning steroids’ antiplasmodial activity could be found in the pertinent literature, despite the fact some steroidal alkaloids having been shown to be active against malaria. *S. nudum* steroidal compounds’ antiplasmodial activity, the good selectivity *in vitro* and it is well-tolerated in early studies in a mouse model support this plant’s potential use in anti-malarial medication and encourage attempts to look for new pharmacophores and more active compounds than diosgenone. Diosgenone hemisynthesis from diosgenin is a good alternative for obtaining this substance when establishing its effects in an infected-mouse model and determining its *in vivo* activity, absorption and toxicity. Preparing other derivatives and synthesizing other compounds involving only diosgenone A- and B-rings could yield new types of antimalarial substances, even though attempting to obtain diosgenone from diosgenin in a single step has failed so far [21]. In short, a type of steroidal saponin whose bioassays in an animal model have shown promissory activity against *P. falciparum* has been reported for the first time here, though its structure should most likely be optimized to achieve the best pharmacokinetic properties. Such types of molecule are obtainable from a cheap, readily available, renewable raw material, such as diosgenin.
